# Supplementary material for: The Dynamic Change in Fatty Acids during the Postharvest Process of Oolong Tea Production
Source: Molecules. 2022 Jul 4;27(13):4298. doi: 10.3390/molecules27134298 (PMC9268070; doi:10.3390/molecules27134298)
Supplement: Supplementary file 1 [file molecules-27-04298-s001.zip › molecules-1787947-Supplementary materials.pdf]

Supplementary Materials

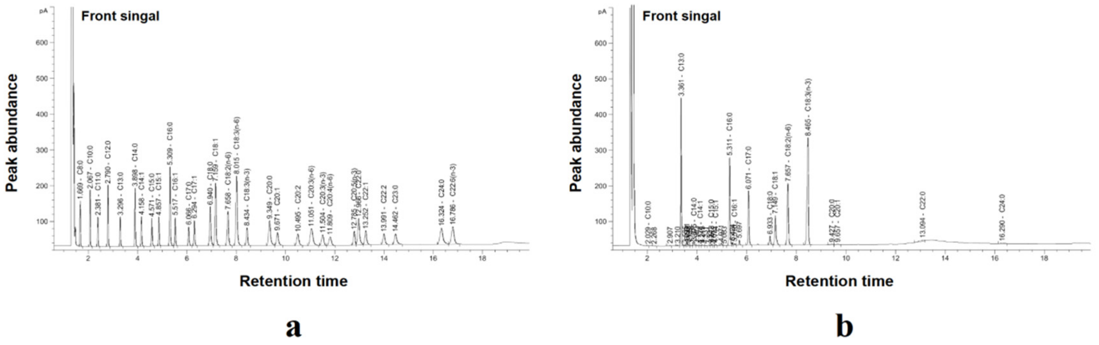

Figure S1. GC-FID electric signal spectrum of fatty acid referencing standards (a) and fresh tea leaf sample (b).

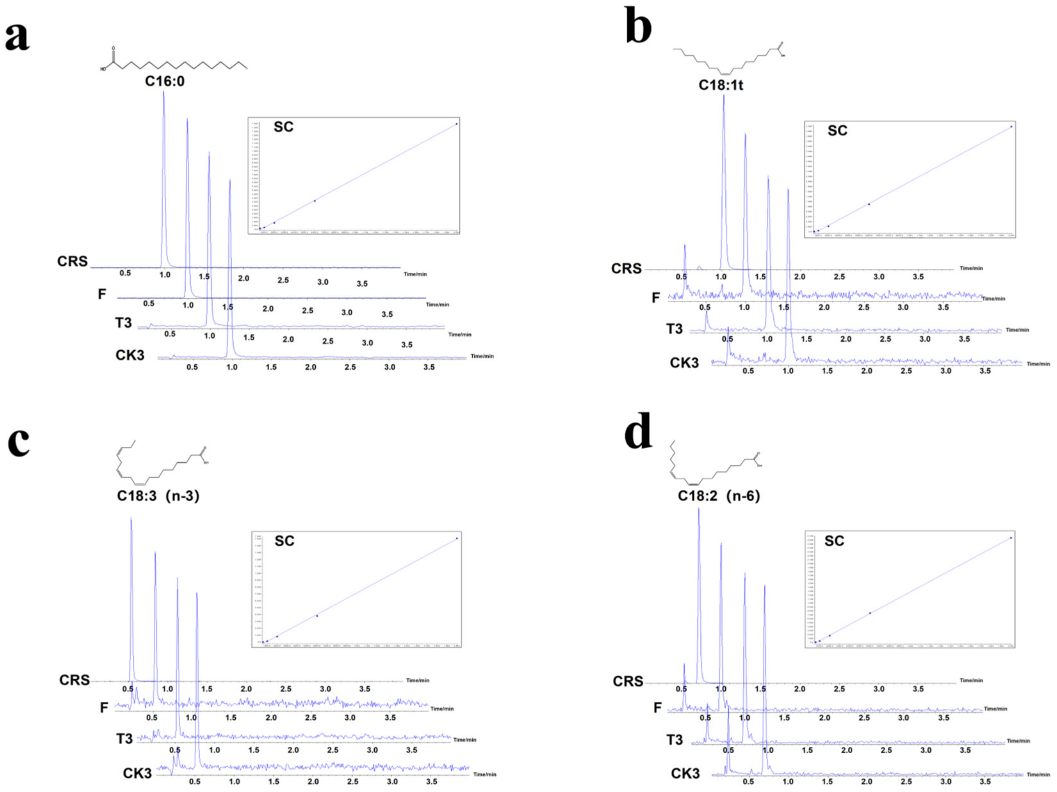

Figure S2. Chromatogram and standard curve of four fatty acids in tea samples during the post-harvest process of oolong tea production production (a) palmitate. (b) trans-9-elaidic acid. (c) α-linolenic acid. (d) linoleic acid; Note: SC: standard curve; CRS: Chemical reference substance; F: fresh leaves; T3: turn-over leaves; CK3: indoor-withered leaves

Table S1. The information of 37 types of fatty acid methyl esters mixed referencing standards

Name	Types	Concentration	CAS
Methyl butyrate	C4:0	400 μg/mL	623-42-7
Methyl hexanoate	C6:0	400 μg/mL	106-70-7
Methyl octanoate	C8:0	400 μg/mL	111-11-5
Methyl decanoate	C10:0	400 μg/mL	110-42-9
Methyl undecanoate	C11:0	200 μg/mL	1731-86-8
Methyl laurate	C12:0	400 μg/mL	29972-79-0
Methyl tridecanoate	C13:0	200 μg/mL	1731-88-0
Methyl myristate	C14:0	400 μg/mL	124-10-7
Methyl myristoleate	C14:1	200 μg/mL	56219-06-8
Methyl pentadecanoate	C15:0	200 μg/mL	7132-64-1
Methyl cis-10-pentadecenoate	C15:1	200 μg/mL	7132-64-1
Methyl palmitate	C16:0	600 μg/mL	90176-52-6
Methyl palmitoleate	C16:1	200 μg/mL	1120-25-8
Methyl heptadecanoate	C17:0	200 μg/mL	1731-92-6
cis-10-Heptadecanoic acid methyl ester	C17:1	200 μg/mL	75190-82-8
Methyl stearate	C18:0	400 μg/mL	112-61-8
trans-9-Elaidic acid methyl ester	C18:1t	200 μg/mL	2462-84-2
cis-9-Oleic acid methyl ester	C18:1c	400 μg/mL	112-62-9
Methyl linolelaidate	C18:2(n-6t)	200 μg/mL	2566-97-4
Methyl linoleate	C18:2(n-6c)	200 μg/mL	112-63-0
Methyl arachidate	C20:0	400 μg/mL	1120-28-1
Methyl γ-linolenate	γ-C18:3	200 μg/mL	7361-80-0
Methyl cis-11-eicosenoate	C20:1 (n-9)	200 μg/mL	2390-09-2
Methyl linolenate	α-C18:3	200 μg/mL	7361-80-0
Methyl heneicosanoate	C21:0	200 μg/mL	6064-90-0
cis-11,14-Eicosadienoic acid methyl ester	C20:2	200 μg/mL	61012-46-2
Methyl behenate	C22:0	400 μg/mL	929-77-1
cis-8,11,14-Eicosatrienoic acid methyl ester	C20:3(n-6)	200 μg/mL	21061-10-9
Methyl erucate	C22:1	200 μg/mL	1120-34-9
cis-11,14,17-Eicosatrienoic acid methyl ester	C20:3(n-3）	200 μg/mL	55682-88-7
cis-5,8,11,14-Eicosatetraenoic acid methyl ester	C20:4	200 μg/mL	2566-89-4
Methyl tricosanoate	C23:0	200 μg/mL	2433-97-8
cis-13,16-Docosadienoic acid methyl ester	C22:2	200 μg/mL	61012-47-3
Methyl lignocerate	C24:0	400 μg/mL	2442-49-1
cis-5,8,11,14,17-Eicosapentaenoic acid methyl ester	C20:5（n-3）	200 μg/mL	2734-47-6
Methyl nervonate	C24:1	200 μg/mL	2733-88-2
cis-4,7,10,13,16,19-Docosahexaenoic acid methyl ester	C22:6（n-3）	200 μg/mL	301-01-9

Note: “m” and “n” indexes of Cm:n represent the number of carbon atoms and unsaturated double bonds in the fatty acid carbon chain, respectively. (n-3), (n-6) and (n-9) indicate that the first double bond of fatty acid starting from the methyl end is between 3 and 4, 6 and 7, 9 and 10, respectively. c，t represent the *cis-* and *trans-* conformations of fatty acids, respectively.

Table S2. Mass spectrometry parameters of four fatty acids

Fatty acid	Product ion （m/z）	Daughter ion （m/z）	DP (V)	CE(V)	CXP(V)
Palmitate	301.1	255.0	-28	-10	-9
(C16:0)	301.1	45	-28.7	-21	-4
trans-9-Elaidic acid	327.1	281.1	-36	-11	-6
(C18:1t)	327.1	45.0	-34	-32	-5
α-linolenic acid	323.0	277.1	-26	-10	-6
(C18:3 (n-3))			-33	-29	-6
linoleic acid	325.2	279.1	-31	-10	-6
(C18:2 (n-6))		45.1	-30	-30	-4.3

DP: declustering potential; CE: collision energy; CXP: collision cell exit potential
